# Assessment of Quality of Life in Men Treated for Infertility in Poland

**DOI:** 10.3390/ijerph19052950

**Published:** 2022-03-03

**Authors:** Marta Makara-Studzińska, Agnieszka Limanin, Agnieszka Anusiewicz, Paula Janczyk, Dorota Raczkiewicz, Anita Wdowiak-Filip, Michał Filip, Iwona Bojar, Krzysztof Lukaszuk, Artur Wdowiak

**Affiliations:** 1 Department of Health Psychology, Institute of Nursing and Midwifery, Faculty of Health Sciences, Jagiellonian University Medical College, ul. Kopernika 25, 31-501 Krakow, Poland; marta.makara-studzinska@uj.edu.pl; 2 FertiMedica—Fertility Center, ul. Jana Pawła Woronicza 31 lok. 8U, 02-640 Warsaw, Poland; agnieszkalimanin@interia.pl; 3 Independent Public Clinical Hospital No. 4 in Lublin, ul. Jaczewskiego 8, 20-954 Lublin, Poland; agnieszkaanusiewicz@wp.pl; 4 Laboratory of Fundamentals of Maternity Care, Institute of Nursing and Midwifery, Faculty of Health Sciences, Jagiellonian University Medical College, ul. Zamoyskiego 58, 30-523 Krakow, Poland; paula.janczyk@uj.edu.pl; 5 Department of Medical Statistics, School of Public Health, Center of Postgraduate Medical Education, Kleczewska 61/63, 01-826 Warsaw, Poland; 6 Department of Cosmetology and Aesthetic Medicine, Medical University of Lublin, ul. Chodźki 1, 20-093 Lublin, Poland; anita.wdowiak@gmail.com; 7 Department of Obstetrics and Pathology of Pregnancy, Medical University of Lublin, ul. Staszica 16, 20-081 Lublin, Poland; michal.a.filip@gmail.com; 8 Department of Women’s Health, Institute of Rural Health in Lublin, ul. Jaczewskiego 2, 20-090 Lublin, Poland; iwonabojar75@gmail.com; 9 Department of Obstetrics and Gynecological Nursing, Faculty of Health Sciences, Medical University of Gdansk, ul. Sklodowskiej-Curie 3a, 80-210 Gdansk, Poland; luka@gumed.edu.pl; 10 Invicta Research and Development Center, ul. Polna 64, 81-740 Sopot, Poland; 11 iYoni App by LifeBite, ul. Martyniaka 16 lok. 1, 10-763 Olsztyn, Poland; 12 Chair of Obstetrics and Gynecology, Faculty of Health Sciences, Medical University of Lublin, 4-6 Staszica St., 20-081 Lublin, Poland; wdowiakartur@gmail.com

**Keywords:** FertiQoL, WHOQoL-BREF, assisted reproductive technologies, infertility, quality of life, intrauterine insemination, in vitro fertilization, men

## Abstract

The aim of this study was to assess the quality of life (QoL) of men treated for infertility in Poland. This cross-sectional study was conducted using the Abbreviated World Health Organization Quality of Life questionnaire (WHOQoL-BREF), Fertility Quality of Life tool (FertiQoL) and an author-constructed questionnaire. The study included 1200 men treated for infertility without the use of assisted reproductive technology (non-ART), intrauterine insemination (IUI) and in vitro fertilization (IVF). The control group consisted of 100 healthy men with confirmed fertility. The quality of life assessed by the WHOQoL-BREF questionnaire was significantly lower in study groups in the Environmental domain, compared to the control group (*p =* 0.009). Statistically significant differences were found in the case of FertiQoL subscales: Emotional, Mind-Body, Relational, and Treatment Environment, depending on applied treatment. Men whose partners were treated without the use of ART assessed their QoL significantly more negatively than those treated with IUI. Reproductive problems and type of their treatment influenced the quality of life of the affected men. Non-ART treatment, rural place of residence, and increased BMI were associated with lower QoL.

## 1. Introduction

Infertility is considered to be a social problem affecting nearly 15% of couples at reproductive age [1]. It is a human right to enjoy the highest attainable state of health, therefore, the inability to have offspring interferes with this right [2]. Partners with a fertility diagnosis are ordered to perform various tests before starting treatment. At the beginning, the semen, the patency of the fallopian tubes, and the ovarian reserve are examined. This allows the couple to choose the appropriate therapy. Depending on the diagnosis, this can be male treatment, ovulation stimulation, intrauterine insemination (IUI) or in-vitro fertilization (IVF). Various countries are implementing various programs to support the generation of offspring among couples with reduced fertility. Between 2016 and 2020 Poland ran only one governmental program to support couples in their way to diagnose infertility. Participation in this program was only possible for couples prior to diagnosis. There was no governmental financial support program for partners during treatment, except for some local initiatives [3]. Consequently, access to full, rapid and professional support may be limited for the less wealthy, or a major constraint for them during treatment. The location of infertility treatment centers in large cities can also be a limitation for residents of non-urban areas [4].

Women with a diagnosis of infertility feel the deterioration of the quality of life (QoL) more strongly and more often than their partners [5]. Due to gender-role expectations, this is particularly expressed in countries with strongly traditional views, where not having an offspring is often associated with seeing the cause in the woman [6]. However, the emotional response to infertility is similar between genders. Males with the diagnosis of infertility experience depression, anxiety [7,8] and stress [9] more often than fertile men. Among men during infertility treatment sexual problems, such as premature ejaculation and other erectile dysfunctions are more common [7], and especially strongly expressed among those in whom the male factor is the cause of infertility [10]. Moreover, in more children-oriented cultures the severity of depression among men affected by infertility is more strongly expressed [10].

Couples undergoing infertility treatment face the need to adjust their relationship to new circumstances and extended treatment time. This results in increased feelings of stress, social pressure, and decreased marital satisfaction. Men in particular experience reduced sexual satisfaction in their relationships [5]. Researchers from Denmark identified increased stress as a factor reducing sperm parameters [11]. Numerous researchers point to the adverse impact of infertility induced stress to subsequent fertility and treatment outcome [12,13]. Better QoL was determined among infertile couples who were characterized by higher social support, self-esteem, marital and sexual satisfaction [14]. The QoL of men treated for infertility depends on a variety of factors, including higher age, education level, monthly income, place of residence, duration of infertility [4,15], and male-factor infertility [16].

Given the above, it is important to find factors that reduce quality of life that are specific of Polish men undergoing infertility treatment, as then, through targeted spousal support programs, an increase in the overall quality of life of men can be achieved [16].

The purpose of this study was to compare the quality of life of men undergoing different infertility treatments. Moreover, the aim was to investigate the socio-demographic determinants of men’s quality of life.

## 2. Materials and Method

### 2.1. Study Groups

This study is the second part of a research project devoted to assessment of the quality of life of patients treated for infertility in Poland. The first part of the project was aimed to evaluate the quality of life of women treated for infertility in Poland and was presented in our previous publication [17].

The study was carried out between January 2017 and June 2018, in the OVUM Medical Centre as well as in the OVEA Obstetric—Gynaecological Office (both in Lublin, Poland).

The Bioethical Committee at the Medical University in Lublin gave its consent for this research, No. KE-0254/306/2016.

Recruitment of study participants was carried out by direct inquiry at the aforementioned clinics. The study was anonymous and voluntary. Consent for study participation was gained after the explanation of its course and purpose. The study groups included men treated with their partners with various methods due to primary and secondary infertility. The WHO classification was adopted as the inclusion criterion, which defines infertility as the inability to achieve pregnancy after one year of regular unprotected intercourse [18].

None of the potential causes of infertility were factors which could disqualify. The female partners of the men participating in the study underwent intrauterine insemination (IUI) or in vitro fertilization (IVF) for the first time or, both female and/or male, were treated without the use of assisted reproductive techniques (non-ART). The IUI was classified as the ART in this study, although some definitions do not include it in the ART. The control group consisted of men with confirmed fertility. Men from the study group completed the questionnaire 24–35 h after pharmacological induction of ovulation in their partners. Men from the control group answered the questions after confirming ovulation in their partners. The efficacy of infertility treatment has not been analyzed. Male partners from the control group had babies conceived without treatment, born 2–4 years earlier. These couples did not plan to have more children. All data were collected using questionnaires. A total of 1200 men who were treated due to infertility with various methods were included to the study as study groups:

- 400 men were treated without using assisted reproductive technology (non-ART);
- 400 men were treated with the intrauterine insemination (IUI);
- 400 men were treated using in vitro fertilization (IVF).

A total of 100 men with confirmed fertility, namely who had children (2–4 years after childbirth) were the control group.

It was assumed right from the start that the studied groups of men treated with IVF, IUI and without ART would be equal in size. During the survey, we were collecting the questionnaires until we had 400 correctly completed questionnaires for each group. The duration of the study was limited by university regulations.

### 2.2. Survey Questionnaire

The study was conducted by the method of a diagnostic survey, with the use of an author-constructed questionnaire, the Abbreviated World Health Organization Quality of Life questionnaire (WHOQoL-BREF) [19], and the Polish version of the Fertility Quality of Life tool (FertiQoL) [20].

Socio-demographic data was obtained using a questionnaire prepared by the authors. The questions included in it concerned age, data related to the place of residence, education, work, income, household, health condition, etc. Before the study, a questionnaire was checked on 30 men in a pilot study. This contributed to the modification of the survey.

Four domains are assessed on WHOQoL-BREF: physical, psychological, social, and environmental. FertiQoL consists of two main parts divided into subscales. The first part is divided into Emotional, Relational, Mind/Body, and Social subscales. The second part is divided into Treatment Tolerability and Treatment Environment subscales. Both questionnaires were described in detail in our previous study aimed to evaluate the quality of life of women treated for infertility in Poland [17].

### 2.3. Statistical Methods

Results for categorical variables were presented as counts (n) and percentages (%). Results for continuous variables were presented as minimum, maximum, mean (M) and standard deviation (SD). Statistical tests were used as follows:

- Pearson’s chi-square test to compare the categorical variables between study groups;
- analysis of variance F test for to compare continuous variables between study groups. If statistical dependence between study groups was found, post hoc analyses were conducted to compare every pair of study groups and the least significant difference test was used.

Multiple regression was used to correlate total score of FertiQoL with characteristics separately in every infertility treatment group.

The significance level was assumed to be 0.05.

SPSS software was used in statistical analyses.

## 3. Results

### 3.1. Respondents’ Characteristics

Table 1 presents the characteristics of the study groups.

The mean age of all the examined men (the control group, the non-ART group, the IUI group and the IVF group) was approximately 35 years and did not significantly differ between the four examined groups.

The respondents from the control group, non-ART, IUI and IVF groups lived most common in urban areas, graduated university, had BMI indicated overweight and their net income per person in a household exceed 2000 PLN a month. The majority of the men treated for infertility had no children. The time in which they have been trying to conceive a child ranged from 1–14 years, approximately 3.1–3.4 years, on average.

### 3.2. Comparison of Quality of Life (WHOQoL-Bref) between Respondents with Infertility and Those Who Had Children

**Figure 1:**
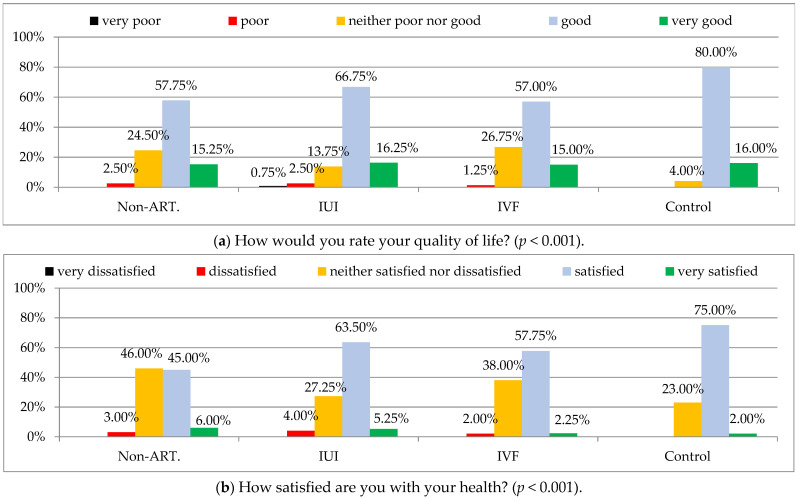
General quality of life (**a**) and health satisfaction (**b**) according to WHOQOL-BREF in study groups. Chi-square test was used. Non-ART—men treated for infertility without the use of assisted reproductive technology; IUI—intrauterine insemination; IVF—in vitro fertilization; WHOQoL-Bref—Abbreviated World Health Organization Quality of Life questionnaire.

Quality of life was evaluated as good or very good by 96% of the men who have already had children, while by significantly lower percentage of the men treated for infertility (*p* < 0.001), (Figure 1a).

In addition, satisfaction with health was evaluated as good or very good by a significantly lower percentage of the men treated for infertility, compared to the men with confirmed fertility (*p* < 0.001), (Figure 1b).

The men in all the examined groups evaluated their physical health lower than the other domains (Table 2). Physical health was assessed significantly lower by the men treated using IVF than by those treated using IUI, or treated without ART. The evaluation of psychological health and social relations did not significantly differ between the study groups (*p* = 0.111 and *p* = 0.060, respectively). Environment was assessed significantly lower by the men treated for infertility (IUI, IVF or without ART) than by those who had children.

### 3.3. Comparison of Quality of Life (Acc. to the FertiQoL) between Methods of Infertility Treatment

**Figure 2:**
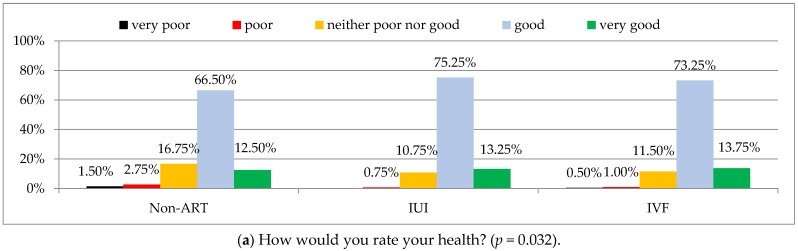

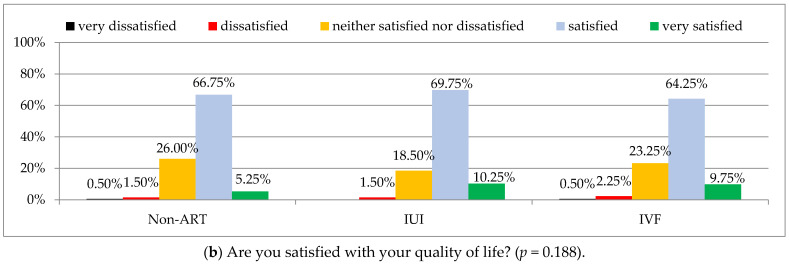
General health (**a**) and quality of life satisfaction (**b**) according to FertiQoL in study groups. Chi-square test was used. Non-ART—men treated for infertility without the use of assisted reproductive technology; IUI—intrauterine insemination; IVF—in vitro fertilization; FertiQoL—Fertility Quality of Life tool.

Health was assessed as good by approximately 2/3 of the men treated for infertility without ART and by a significantly higher percentage of the men treated using IUI or IVF (approximately ¾), (Figure 2a).

Satisfaction with the quality of life was assessed most commonly as good (by 2/3 of the men treated for infertility) and no significant difference between three methods of infertility treatment (non-ART treatment, IUI or IVF) was found (*p* = 0.188), (Figure 2b).

The overall quality of life according to FertiQoL was assessed significantly lower by the men treated for infertility without ART than those treated using IUI, while it did not significantly differ between the men treated with IVF and IUI, nor between the men treated with IUI and without ART (Table 3). The same applied to Core FertiQoL.

Emotional, Mind/Body and Relational domains of the quality of life were assessed significantly lower by the men treated without ART than by those treated with IUI.

Mind/Body and Relational domains of the quality of life were assessed significantly lower by the men treated with IVF than by those treated with IUI.

Emotional domain of the quality of life was assessed significantly lower by the men treated without ART than by those treated with IVF.

The assessment of social domain of the quality of life did not differ significantly between the men treated using the three methods (*p* = 0.099), the same applied to the treatment in general (*p* = 0.255), and to the tolerability of treatment (*p* = 0.201). However, the environment of treatment was assessed significantly lower by the men treated using IUI or non-ART than those treated with IVF.

### 3.4. Correlations between Characteristics of Study Groups and Quality of Life (Acc. to FertiQoL)

Table 4 presents the results of regression analyses of total FertiQoL scores against the characteristics of the men treated using the three analyzed methods of infertility treatment (non-ART, IUI, IVF).

BMI significantly lowered the overall quality of life score in the three analyzed groups of the men treated for infertility without ART, using IUI or IVF.

In the group of the men treated for infertility without ART, the total quality of life score was significantly lower among the men living in rural areas than among those living in towns. It was also significantly lower among the men with tertiary education than with basic vocational or secondary education, as well as lower among the men with shift or flexible working hours than among the men with fixed working hours. Moreover, among the men treated for infertility without ART, the lower the total quality of life score, the longer the time trying for a baby, on average.

In the group of the men treated for infertility using IUI, the total quality of life score was significantly lower among the men living in rural areas than among those living in towns. The lower the total quality of life score, the younger the men and the longer the time trying for a baby, on average.

In the group of the men treated for infertility using IVF, the total quality of life score was significantly lower among the men living in rural areas than in those living in cities. It was also significantly lower among the men with manual or mixed job than among the men with non-manual job. In the group of the men treated for infertility using IVF, the lower the total quality of life score, the older the men, on average.

The total quality of life score did not correlate with having children and with income per capita in the three analyzed groups of the men treated for infertility without ART, using IUI or IVF.

## 4. Discussion

In the present study, quality of life was assessed using WHOQOL-BREF and FertiQoL questionnaires. Cross-group comparisons by treatment type were performed (non-Art., IUI, IVF). The possible influence of group characteristics on the mean FertiQoL score was evaluated.

The mean age of the examined men was 35. The majority of the respondents lived outside the city, did not have children, and the mean duration of their infertility was approximately 3 years.

No significant differences in the quality of life and satisfaction with the state of health assessed using the WHOQOL-BREF were observed between men treated due to infertility and those from the control group with confirmed fertility in the Physical, Psychological and Social subscales. A significant difference between the study groups and the control group was found in the Environment domain. This relationship was not noted between the examined women [17]. Bolsoy et al. also reported that in the Environment domain there occurred similar differences in the presented results between men and women [21]. The items within the Environment domain focus on the assessment of the financial situation, access to health care and information, as well as on the environment of life. This is coherent with the findings of Keramat et al. [14], where lower income was associated with deterioration in QoL in Environmental subscale. Poland as a country does not provide universal services for fertility, nor treatment is included in insurance coverage. Impact of financial status, education level and residual area were proven to be important QoL factors in numerous studies [4,5,14,20,21].

This publication is the second part of the study on the quality of life of patients treated for infertility in Poland [17]. In the first part, we the quality of life of women, and compared QoL according to the treatment method. Among the examined women significant differences in the quality of life were found only in the Emotional domain (FertiQoL). Women treated with IUI had the lowest emotional score (53.8 ± 19.5). Among the men studied, a much greater diversity of results was observed.

In the systematic review concerning gender differences in experiencing infertility, it was found that men experience the effects of treatment to a lower degree, and among them depression and mood disorders occur more rarely than in their female partners [5]. In the presented study the difference between genders was also observed. In each of examined groups women scored lower Core FertiQoL than men (non-Art.: 61.9 ± 14.1 vs. 67.8 ± 13.1; IUI: 62.8 ± 13.4 vs. 71.1 ± 13.1 and IFV 63.8 ± 14.5 vs. 68.5 ± 12.0). Women are attributed a greater experience of deteriorating quality of life [21]. The majority of medical procedures necessary to achieve pregnancy are performed on women, and they also bear the mental load of maintaining pregnancy. Therefore, they may impose on themselves higher expectations with respect to treatment, and perceive greater stress and responsibility, compared to men [22,23,24].

In this study an overall Core Fertiqol score for men was 68.8 for non-Art., 69.8 for IVF, and 71.0 for IUI. In other studies, the reported results were 83.3 [15], 60.6 [25], and 69.9 [16]. Warchol-Biedermann conducted a study among males treated for infertility in another large town in Poland [26]. The lowest Core FertiQoL score was 75.23, while the highest score obtained by men in this study was 71.1. The only category where the results were similar was the Emotional domain. The remaining differences may be explained by the fact that men in the study by Warchol–Bidermann were by 4 years younger, on average than in the current study (30.24 vs. 35.0), and were before their first cycle of infertility treatment. In addition, the majority (85%) of men examined by Warchol lived in urban areas, whereas in this study approx. 40% lived in a city. The place of residence in a large city positively correlated with the declared level of the quality of life [16,27]. Men examined by Warchol–Biedermann started treatment for the first time, and during the treatment cycle the level of their quality of life decreased [26]. The men in the presented study group were before starting the treatment cycle; however, this was not necessarily their first cycle. Numerous scientific studies show that the quality of life of men and women decreases with the duration of reproductive problems [14,28,29].

In a study by Asazawa an overall level of FertiQoL was similar to the results presented in the current study (69.9 vs. 68.8) [16]. While analyzing the results between individual categories it was noted that they differ by even 15 scores. In studies concerning the quality of life of patients treated due to infertility analyses are performed according to many factors, e.g., income, education level, type of infertility [30], duration of infertility, number of treatment cycles, and place of residence [27]. In the present study we additionally tried to evaluate if different forms of treatment might potentially have had an influence on QoL in Polish men. The group who received IUI treatment, which obtained the highest FertiQoL score (71.0 ± 11.2), was compared with the non-Art. group (68.8 ± 11.3, 0.008), where the result was the lowest, the difference occurred to be significant. In the presented study the IUI group obtained the highest results in all Core FertiQoL categories, whereas the non-Art. group obtained the lowest scores, and in all categories the difference between these groups was significant. Significant differences between the IUI and IFV groups were also observed in the Mind/Body and Relational domains. Jahromi et al. reported that the type of treatment did not play a significant role in their research [30]. Numerous scientific reports prove that the experience of problems with getting pregnant is influenced by factors such as race, ethnicity, religion, and social class [31]. This biopsychosocial theory of infertility was also promoted in other study, where a cross-cultural comparison between three countries was conducted [32]. The researchers concluded that the differences between study results should not be investigated as differences between countries, but rather exclusively in the specific cultural context on the subgroup and individual levels within a given society [32].

Considering the above it should be pointed out that the presented study the result in the Social domain was equally low for all groups. The Social domain assesses the perception of social pressure concerning having children, the sense of being inferior, lack of understanding in a family, the feeling of isolation, and the perceived level of support by acquaintances. The Roman Catholic Church, which dominates in Poland, does not accept the use of IUI and IVF, which builds a sense of guilt in people treated with these methods [33]. In addition, in more children-oriented cultures, the feeling of inadequacy, and the sense of guilt enhanced by being childless lead to social isolation [34].

In their study, Aarts et al. [35] and Sexty et al. [32] age positively correlated with the overall FertiQoL score. In the conducted study, the quality of life increased with the age of men treated with IUI, but not in the rest of the respondents. Sexty et al. assumed that issues related to education may translate into type of profession and the financial situation, and thus shape the quality of life [32]. However, men treated with IVF did not present those results. They presented lowest scores in social scale. Primary infertility, prolonged infertility time, less social support and experiencing social stigma by not presenting with child may contribute to lower QoL [4,5,9,14,16].

The majority of respondents from the non-Art. Group, who scored lowest, had a low level of income. Therefore, the question may be posed whether income exerts an effect on the quality of life. However, in this research the total quality of life score did not correlate with income per capita in all of the analyzed groups. The reports to-date from qualitative studies suggest that a high cost of treatment is one of the main difficulties mentioned by couples undertaking treatment [36]. The quality of life was significantly lower in men living in rural areas than in urban areas in all groups. It can be assumed, given the literature [4,14,37], that this may be due to the lower economic status and lower education of the inhabitants of Polish villages compared to people from the cities; however, those factors did not play a significant role in the presented group. Therefore, considering the fact that the WHOQOL Environmental scores were lower than the control, this could rather be explained by remaining WHOQOL components. These are: availability of health centers and quality of treatment, the need to travel to medical appointments and examinations, access to information, the possibility of pursuing interests [19].

The higher BMI scores negatively correlated with the total quality of life score in all the analyzed groups. Obesity and overweight have been found to be a risk factor in the development of depression. It is related to the lack of acceptance of your own appearance. Altered neurotransmitter conduction in the body and hormone levels cause the exhibition of abnormal emotions and behaviors, such as anxiety or specific phobia [38].

Despite our best efforts, the research is not without limitations. Firstly, from among all Polish clinics of infertility treatment only two were selected by convenience sampling, instead of random sampling. Thus, the possibility that the QoL may be biased cannot be completely ruled out. Furthermore, the reason why certain differences occurred between the study groups have not been investigated. The examination could be improved by analysis of other variables including depression syndromes and causes of infertility among patients.

Another limitation of our study is the lack of information about the specific male treatment and infertility type. The male infertility factor was not considered in the study because it does not have a specific definition. We did not include the male infertility factor in the study because it does not have a clear definition. Classification of extremely low semen parameters as a male infertility factor is obvious, however setting an exact limit when men can be qualified as responsible for infertility will always be imperfect. In addition, spermiogram standards changed in 2021, and sperm parameters may vary from extremely low to normal even in healthy men. The selection of the method of treatment is dictated by many factors, especially medical history and state of health. It should be kept in mind that the quality of life may affect the outcome of treatment, if only by an increased life stress as a factor reducing men’s fertility, e.g., sperm parameters [11]. In order to know which persons are particularly exposed to a decrease in the quality of life local studies should be carried out, focused on individuals. The obtained results of the conducted study showed the complicated situation of patients treated for infertility in Poland. As almost one in five couples of childbearing age in this country have reproductive problems, this creates a need for support for these people. This undoubtedly results in the need for further research on this problem.

## 5. Conclusions

1. The quality of life of men treated due to infertility is affected by their reproductive problems.
2. The type of treatment had an impact on quality of life in men who were undergoing infertility treatment.
3. Rural place of residence, increased BMI, non-ART treatment has a negative impact on quality of life of men treated for infertility.
4. Attention should be paid to health, psychological, and environmental factors which deteriorate the quality of life of male patients undergoing infertility treatment, because stress associated with a low quality of life may result in worse outcomes of treatment.

## Figures and Tables

**Table 1:** Characteristics of the study groups.

Variable, Parameter	IU or Category	Infertility Treated Men			Control Group (N = 100)	Comparison between Groups *p*
		Non-ART (N = 400)	IUI (N = 400)	IVF (N = 400)		
Age, Min–Max M ± SD	years	24–52 34.9 ± 4.5	25–54 35.0 ± 5.0	26–53 35.6 ± 4.3	25–51 34.7 ± 4.6	0.104
Place of residence, *n* (%)	city	154 (38.50)	162 (40.50)	149 (37.25)	51 (51.00)	0.055
	town	120 (30.00)	97 (24.25)	98 (24.50)	22 (22.00)	
	rural area	126 (31.50)	141 (35.25)	153 (38.25)	27 (27.00)	
Level of education, *n* (%)	basic vocational	63 (15.75)	40 (10.00)	46 (11.50)	16 (16.00)	0.065
	secondary	128 (32.00)	152 (38.00)	132 (33.00)	40 (40.00)	
	tertiary	209 (52.25)	208 (52.00)	222 (55.50)	44 (44.00)	
BMI, Min–Max M ± SD	kg/m^2^	21.4–36.2 27.2 ± 2.8	16.9–38.1 27.0 ± 3.6	17.3–42.9 27.1 ± 2.9	16.7–36.2 27.5 ± 3.8	0.540
BMI, *n* (%)	underweight	0 (0.00)	7 (1.75)	2 (0.50)	3 (3.00)	<0.001
	normal weight	102 (25.00)	103 (25.75)	80 (20.00)	21 (21.00)	
	overweight	230 (57.50)	216 (54.00)	267 (66.75)	51 (51.00)	
	obesity	68 (17.00)	14 (18.50)	51 (12.75)	25 (25.00)	
Having children, *n* (%)	yes, from the current relationship	20 (5.00)	40 (10.00)	30 (7.50)	100 (100.00)	0.039 *
	yes, from a previous relationship	26 (6.50)	25 (6.25)	16 (4.00)		
	no	354 (88.50)	335 (83.75)	354 (88.50)	0 (0.00)	
Time trying to conceive a child, Min–Max M ± SD	years	1–10 3.2 ± 1.9	1–9 3.1 ± 1.9	1–14 3.4 ± 2.0	-	0.219
Type of job, *n* (%)	manual	130 (32.50)	98 (24.50)	126 (31.50)	32 (32.00)	0.069
	non-manual	151 (37.75)	145 (36.25)	146 (36.50)	33 (33.00)	
	mixed	119 (29.75)	157 (39.25)	128 (32.00)	35 (35.00)	
Working hours, *n* (%)	fixed	160 (40.00)	143 (35.75)	147 (36.75)	36 (36.00)	<0.001
	shift work	148 (37.00)	117 (29.25)	151 (37.75)	26 (26.00)	
	flexible	92 (23.00)	140 (35.00)	102 (25.50)	38 (38.00)	
Monthly net income per 1 person in a household (thousand PLN), *n* (%)	below 1	45 (11.25)	27 (6.75)	36 (9.00)	2 (2.00)	<0.001
	1–1.5	109 (27.25)	94 (23.50)	119 (29.75)	21 (21.00)	
	1.5–2	119 (29.75)	102 (25.50)	120 (30.00)	23 (23.00)	
	above 2	127 (31.75)	177 (44.25)	125 (31.25)	54 (54.00)	

* without control group, Chi-square test for categorical variables or F test for quantitative variables. M—mean; SD—standard deviation; BMI—body mass index; PLN—Polish currency—Polish złoty; non-ART—men treated for infertility without the use of assisted reproductive technology; IUI—intrauterine insemination; IVF—in vitro fertilization.

**Table 2:** WHOQOL-BREF scores in study groups.

Domain	Infertility Treated Men			Control Group (N = 100)	Comparison between Groups *p*	*p* for Post Hoc Tests					
	Non-ART (N = 400)	IUI (N = 400)	IVF (N = 400)			Non-ART vs. Control	IUI vs. Control	IVF vs. Control	Non-ART vs. IUI	Non-ART vs. IVF	IUI vs. IVF
	M ± SD										
Physical health	56.0 ± 7.6	55.4 ± 7.8	54.3 ± 6.2	55.7 ± 8.6	0.015	0.737	0.737	0.099	0.288	0.002	0.038
Psychological	66.0 ± 8.4	66.7± 7.5	67.0 ± 8.5	68.0 ± 7.4	0.111	n/a	n/a	n/a	n/a	n/a	n/a
Social relationships	69.3 ± 11.1	71.0 ± 11.3	71.0 ± 12.5	71.8 ± 11.7	0.060	n/a	n/a	n/a	n/a	n/a	n/a
Environment	69.4 ± 7.2	68.4 ± 7.2	68.5 ± 8.7	71.1 ± 7.3	0.009	0.050	0.002	0.004	0.085	0.129	0.841

F test for analysis of variance was used. The least significant difference test was used as a post hoc test. M—mean; SD—standard deviation; non-ART—men treated for infertility without the use of assisted reproductive technology; IUI—intrauterine insemination; IVF—in vitro fertilization; WHOQOL-BREF—Abbreviated World Health Organization Quality of Life questionnaire, n/a—not applicable.

**Table 3:** FertiQoL scores in study groups.

Domain	Infertility Treated Men			Comparison between Groups *p*	*p* for Post Hoc Tests		
	Non-ART (N = 400)	IUI (N = 400)	IVF (N = 400)		Non-ART vs. IUI	Non-ART vs. IVF	IUI vs. IVF
	M ± SD						
Total FertiQoL	68.8 ± 11.3	71.0 ±11.2	69.8 ± 11.8	0.029	0.008	0.204	0.162
Core FertiQoL	67.8 ± 13.1	71.1 ± 13.1	68.5 ± 12.0	0.001	<0.001	0.471	0.004
Emotional	64.2 ± 17.1	67.9 ± 17.6	66.5 ± 14.9	0.006	0.001	0.050	0.217
Mind/body	69.4 ± 17.7	73.6 ± 17.2	70.2 ± 15.9	0.001	<0.001	0.504	0.005
Relational	72.2 ± 16.7	76.2 ± 16.0	72.9 ± 15.6	0.001	<0.001	0.523	0.004
Social	65.6 ± 14.6	66.6 ± 14.9	64.4 ± 13.7	0.099	n/a	n/a	n/a
Treatment FertiQoL	69.8 ± 12.2	70.9 ±11.7	71.2 ± 13.8	0.255	n/a	n/a	n/a
Environment	64.5 ± 13.5	64.8 ± 13.9	67.2± 13.8	0.009	0.788	0.005	0.012
Tolerability	75.1 ± 15.8	77.0 ± 16.7	75.2 ± 18.5	0.201	n/a	n/a	n/a

F test for analysis of variance was used. The least significant difference test was used as a post hoc test. M—mean; SD—standard deviation; non-ART—men treated for infertility without the use of assisted reproductive technology; IUI—intrauterine insemination; IVF—in vitro fertilization; FertiQoL—Fertility Quality of Life tool, n/a—not applicable.

**Table 4:** Multivariable regression analysis results of total FertiQoL scores against characteristics of study groups.

Covariate	IU or Category	Infertility Treated Men					
		Non-ART (N = 400)		IUI (N = 400)		IVF (N = 400)	
		b	*p*	b	*p*	b	*p*
Age	years	0.12	0.360	0.35	0.002	−0.44	0.001
Place of residence	city	−1.01	0.204	1.17	0.125	1.92	0.018
	town	2.54	0.002	2.61	0.001	1.90	0.313
	rural area	reference					
Level of education	basic vocational or secondary	1.81	0.015	1.70	0.007	0.64	0.350
	tertiary	reference					
BMI	kg/m^2^	−0.60	0.005	−0.80	<0.001	−0.58	0.004
Having children	yes	0.03	0.974	0.35	0.635	−0.46	0.630
	no	reference					
How long trying for a baby	years	−0.69	0.024	−1.08	<0.001	−0.27	0.391
Type of job	non-manual	reference					
	manual or mixed	−0.59	0.439	0.73	0.270	−2.81	<0.001
Working hours	fixed	1.63	0.010	0.97	0.096	−0.81	0.229
	shift or flexible	reference					
Monthly net income per 1 person in a household (thousand PLN)	below 1.5	reference					
	1.5–2	1.20	0.138	1.22	0.133	0.33	0.691
	above 2	−0.17	0.838	−0.57	0.429	0.20	0.816

General linear regression was used. b—regression slope term i.e., mean change in total FertiQoL scores per unit of a covariate; BMI—body mass index; PLN—Polish currency—Polish złoty; non-ART—men treated for infertility without the use of assisted reproductive technology; IUI—intrauterine insemination; IVF—in vitro fertilization; FertiQoL—Fertility Quality of Life tool.

## Data Availability

The datasets generated during the current study are available from the corresponding author on reasonable request.

## References

[1] Sun H., Gong T.T., Jiang Y.T., Zhang S., Zhao Y.H., Wu Q.J. (2019). Global, Regional, and National Prevalence and Disability-Adjusted Life-Years for Infertility in 195 Countries and Territories, 1990–2017: Results from a Global Burden of Disease Study, 2017. Aging.

[2] Zegers-Hochschild F., Dickens B.M., Dughman-Manzur S. (2013). Human Rights to in Vitro Fertilization. Int. J. Gynecol. Obstet..

[3] Ministerstwo Zdrowia Program Kompleksowej Ochrony Zdrowia Prokreacyjnego w Polsce w Latach 2016–2020. http://www.archiwum.mz.gov.pl/wp-content/uploads/2018/01/2017-program-kompleksowej-ochrony-zdrowia-prokreacyjnego-nowelizacja-24072017.pdf.

[4] Namdar A., Naghizadeh M.M., Zamani M., Yaghmaei F., Sameni M.H. (2017). Quality of Life and General Health of Infertile Women. Health Qual. Life Outcomes.

[5] Ying L.Y., Wu L.H., Loke A.Y. (2015). Gender Differences in Experiences with and Adjustments to Infertility: A Literature Review. Int. J. Nurs. Stud..

[6] Petok W.D. (2006). The Psychology of Gender-Specific Infertility Diagnoses. Infertility Counseling: A Comprehensive Handbook for Clinicians.

[7] Gao J., Zhang X., Su P., Liu J., Shi K., Hao Z., Zhou J., Liang C. (2013). Relationship between Sexual Dysfunction and Psychological Burden in Men with Infertility: A Large Observational Study in China. J. Sex. Med..

[8] Ahmadi H., Montaser-Kouhsari L., Nowroozi M.R., Bazargan-Hejazi S. (2011). Male Infertility and Depression: A Neglected Problem in the Middle East. J. Sex. Med..

[9] Peronace L.A., Boivin J., Schmidt L. (2007). Patterns of Suffering and Social Interactions in Infertile Men: 12 Months after Unsuccessful Treatment. J. Psychosom. Obstet. Gynecol..

[10] Wischmann T., Thorn P. (2013). (Male) Infertility: What Does It Mean to Men? New Evidence from Quantitative and Qualitative Studies. Reprod. Biomed. Online.

[11] Nordkap L., Jensen T.K., Hansen Å.M., Lassen T.H., Bang A.K., Joensen U.N., Jensen M.B., Skakkebæk N.E., Jørgensen N. (2016). Psychological Stress and Testicular Function: A Cross-Sectional Study of 1215 Danish Men. Fertil. Steril..

[12] Boivin J., Schmidt L. (2005). Infertility-Related Stress in Men and Women Predicts Treatment Outcome 1 Year Later. Fertil. Steril..

[13] Rooney K.L., Domar A.D. (2018). The Relationship between Stress and Infertility. Dialogues Clin. Neurosci..

[14] Keramat A., Masoumi S.Z., Mousavi S.A., Poorolajal J., Shobeiri F., Hazavehie S.M.M. (2014). Quality of Life and Its Related Factors in Infertile Couples. J. Res. Health Sci..

[15] Santoro N., Eisenberg E., Trussell J.C., Craig L.B., Gracia C., Huang H., Alvero R., Casson P., Christman G., Coutifaris C. (2016). Fertility-Related Quality of Life from Two RCT Cohorts with Infertility: Unexplained Infertility and Polycystic Ovary Syndrome. Hum. Reprod..

[16] Asazawa K., Jitsuzaki M., Mori A., Ichikawa T., Shinozaki K., Porter S.E. (2019). Quality-of-Life Predictors for Men Undergoing Infertility Treatment in Japan. Jpn. J. Nurs. Sci..

[17] Wdowiak A., Anusiewicz A., Bakalczuk G., Raczkiewicz D., Janczyk P., Makara-Studzińska M. (2021). Assessment of Quality of Life in Infertility Treated Women in Poland. Int. J. Environ. Res. Public Health.

[18] Zegers-Hochschild F., Adamson G.D., de Mouzon J., Ishihara O., Mansour R., Nygren K., Sullivan E., Vanderpoel S. (2009). International Committee for Monitoring Assisted Reproductive Technology (ICMART) and the World Health Organization (WHO) revised glossary of ART terminology, 2009. Fertil. Steril..

[19] Jaracz K., Kalfoss M., Górna K., Bączyk G. (2006). Quality of life in Polish respondents: Psychometric properties of the Polish WHOQOL–Bref. Scand. J. Caring Sci..

[20] Boivin J., Takefman J., Braverman A. (2011). The Fertility Quality of Life (FertiQoL) tool: Development and general psychometric properties. Fertil. Steril..

[21] Bolsoy N., Taspinar A., Kavlak O., Sirin A. (2010). Differences in Quality of Life between Infertile Women and Men in Turkey. J. Obstet. Gynecol. Neonatal Nurs. JOGNN.

[22] Karaca A., Unsal G. (2015). Psychosocial Problems and Coping Strategies among Turkish Women with Infertility. Asian Nurs. Res..

[23] Galhardo A., Cunha M., Pinto-Gouveia J., Matos M. (2013). The Mediator Role of Emotion Regulation Processes on Infertility-Related Stress. J. Clin. Psychol. Med. Settings.

[24] Cserepes R.E., Kollár J., Sápy T., Wischmann T., Bugán A. (2013). Effects of Gender Roles, Child Wish Motives, Subjective Well-Being, and Marital Adjustment on Infertility-Related Stress: A Preliminary Study with a Hungarian Sample of Involuntary Childless Men and Women. Arch. Gynecol. Obstet..

[25] Hsu P.Y., Lin M.W., Hwang J.L., Lee M.S., Wu M.H. (2013). The Fertility Quality of Life (FertiQoL) Questionnaire in Taiwanese Infertilecouples. Taiwan. J. Obstet. Gynecol..

[26] Warchol-Biedermann K. (2021). The Etiology of Infertility Affects Fertility Quality of Life of Males Undergoing Fertility Workup and Treatment. Am. J. Men’s Health.

[27] Wadadekar G., Inamdar D., Nimbargi V. (2021). Assessment of Impact of Infertility & Its Treatment on Quality of Life of Infertile Couples Using Fertility Quality of Life Questionnaire. J. Hum. Reprod. Sci..

[28] Agostini F., Monti F., Andrei F., Paterlini M., Palomba S., La Sala G.B. (2017). Assisted Reproductive Technology Treatments and Quality of Life: A Longitudinal Study among Subfertile Women and Men. J. Assist. Reprod. Genet..

[29] Chachamovich J.R., Chachamovich E., Ezer H., Fleck M.P., Knauth D., Passos E.P. (2010). Investigating Quality of Life and Health-Related Quality of Life in Infertility: A Systematic Review. J. Psychosom. Obstet. Gynaecol..

[30] Jahromi B.N., Mansouri M., Forouhari S., Poordast T., Salehi A. (2018). Quality of Life and Its Influencing Factors of Couples Referred to An Infertility Center in Shiraz, Iran. Int. J. Fertil. Steril..

[31] Greil A.L., Slauson-Blevins K., McQuillan J. (2010). The Experience of Infertility: A Review of Recent Literature. Sociol. Health Illn..

[32] Sexty R.E., Hamadneh J., Rösner S., Strowitzki T., Ditzen B., Toth B., Wischmann T. (2016). Cross-Cultural Comparison of Fertility Specific Quality of Life in German, Hungarian and Jordanian Couples Attending a Fertility Center. Health Qual. Life Outcomes.

[33] Korolczuk E. (2014). Niepłodność, Tożsamość, Obywatelstwo. Analiza Społecznej Mobilizacji Wokół Dostępu Do in Vitro w Polsce. Etnogr. Biomed..

[34] Makara-Studzinśka M., Wdowiak A., Bakalczuk G., Bakalczuk S., Krys K. (2012). Emotional Problems among Couples Treated for Infertility [Problemy Emocjonalne Wśród Par Leczonych z Powodu Niepłodności]. Seksuologia Pol..

[35] Aarts J.W.M., van Empel I.W.H., Boivin J., Nelen W.L., Kremer J.A.M., Verhaak C.M. (2011). Relationship between Quality of Life and Distress in Infertility: A Validation Study of the Dutch FertiQoL. Hum. Reprod..

[36] Banerjee S., Mary M.N. (2020). Exploring Quality of Life and Perceived Experiences among Couples Undergoing Fertility Treatment in Western India: A Mixed Methodology. Int. J. Curr. Res. Rev..

[37] Sexty R.E., Griesinger G., Kayser J., Lallinger M., Rösner S., Strowitzki T., Toth B., Wischmann T. (2018). Psychometric Characteristics of the FertiQoL Questionnaire in a German Sample of Infertile Individuals and Couples. Health Qual. Life Outcomes.

[38] Barry D., Pietrzak R.H., Petry N.M. (2008). Gender Differences in Associations between Body Mass Index and DSM-IV Mood and Anxiety Disorders: Results from the National Epidemiologic Survey on Alcohol and Related Conditions. Ann. Epidemiol..

